# Implementation of lean Six-Sigma project in enhancing health care service quality during COVID-19 pandemic

**DOI:** 10.3934/publichealth.2021056

**Published:** 2021-10-22

**Authors:** Muhammad Mutasim Billah Tufail, Muhammad Shakeel, Faheem Sheikh, Nuzhat Anjum

**Affiliations:** 1 Department of Management Studies Bahria University Karachi Campus, Pakistan; 2 Department of Business Studies Bahria University Karachi Campus, Pakistan; 3 Pediatric Cardiology Department, NICVD Karachi, Pakistan; 4 Medical Practitioner Karachi, Pakistan

**Keywords:** Lean Six Sigma, LSS project, DMAIC, Kano model, COVID-19, hospital management

## Abstract

The recent outbreak of coronavirus (COVID-19) pandemic has exposed the weakness of the existing healthcare facilities in developing countries, and Pakistan has no exception. The increasing amount of patients has made this condition more vulnerable to failure. It became difficult for health care management to handle the surge of patients. This case study is based on the XYZ hospital system of Pakistan. The hospital initiates passive immunization as a savior in the absence of a vaccine. The process initiates numerous challenges as the same facility was using for passive immunization and routine operations of the hospital. DMAIC lean sig-sigma problem-solving methodology has been adopted to Define, Measure, Analyze, Implement and Control the improvement process for smooth special and routine activities. The staff and patients were interviewed, their issues were listed, and a comprehensive solution was suggested to deal with operational uncertainties. The results identified various factors through VOC and SIPOC processes, prioritized using fishbone diagram, analyzed through Kano model, and finally proposed process improvement by incorporating Kaizen process improvement methodology. Other industries could use this set of tools to evaluate and optimize routine problems, which ultimately enhances the quality and reduces cost.

## 1. Introduction

Healthcare is a unique service industry, and it deals with complex tasks. The COVID-19 pandemic has posed enormous challenges to the health care system globally. Coronavirus disease (COVID-19) caused by severe acute respiratory syndrome coronavirus 2 (SARS-CoV-2) is an infectious disease [1]. After its outbreak in Wuhan, China (December 2019), the virus's rapid spread sparked alarm worldwide. The World Health Organization declared this outbreak a pandemic, and countries worldwide are grappling with a surge in confirmed cases. As of August 6, 2021, over 200 million documented cases of COVID-19 have been reported, resulting in more than 4.2 million deaths in 215 countries [2]. As the virus spreads through close contact and by tiny droplets produced during cough, sneeze, or talk [1],[3], it is essential to maintain adequate social distancing to prevent the spread of the virus [4]. Most countries have responded with preventive measures through health advocacy campaigns, lockdowns, and restricting public gatherings. The hospitals are ramping up their capabilities to care for increasing numbers of infected patients. However, accommodating COVID-19 patients with regular patients will not only increase the number of infections but also create a threat for those who are already suffering from other chronic diseases. To deal with the challenge, the XYZ hospital of Pakistan adopted a lean sig-sigma problem-solving methodology to investigate the possible solution for smooth routine and special COVID-19 patients with optimum quality. Lean principles and tools have been used in Healthcare to tackle principal causes of inefficiency (i.e., waste), increase the quality of the services being provided and reduce costs [5],[6]. The literature reveals the potentials of Lean tools in Healthcare worldwide [7]. Clearly, it highlights its benefits, mainly in terms of increased added value and quality [8], and reductions in waiting time, errors, and costs [9]. Moreover, Lean is said to induce various intangible positive impacts, such as increased customer satisfaction, employee motivation and commitment [10],[11], and even enhanced safety [12].

In this light, the article presents and discusses the key features and results of a Lean Six Sigma (LSS) project developed in Pakistani hospital. The case study is developed adopting an action research approach. The article's aims are fivefold: THE DMAIC, five step process/quality improvement methodology, is adopted. The application of DMAIC in a healthcare organization provides guidelines on how to handle a quality service system toward patient satisfaction [13]. This approach also helps healthcare service providers to reduce waste, variation, and work imbalance in the service processes.

## 2. Lean Six Sigma and its implementation in healthcare industry

The objective of lean Six Sigma is to reduce non-value adding activities, called waste, and reduce process variability. It redesigns business processes by merging two known philosophies, lean and Six Sigma. In 1980, Motorola was the first organization that adopted the Six Sigma methodology [14],[15]. Many other organizations, including Ford, Sony, Kodak, Allied Signal, have incorporated Six Sigma for reducing variability from their production process [16]. The philosophy of lean is based on waste reduction [17], while Six Sigma focuses on precession and accuracy [18]. The TPS (Toyota production system) has incepted the lean methodology, which focuses on waste reduction, efficiency enhancement, quality maximization, and customer satisfaction [19],[20]. Six Sigma process improvement methodology is based on the DMAIC approach, Define Measure, Analyze, Improve and control [21]. In order to enhance customer satisfaction, this paper tends to adopt the LSS methodology in improving the health sector overall experience.

The global health care sector has widely adopted Six Sigma methodology because of its zero-tolerance mechanism, which helps minimize medical procedural errors [22]–[24]. In the meantime, several issues, including procedural errors, excessive cost of operation, resource management, and quality optimization, can be optimized by proper implementation of lean technique [25]–[28]. [29] have argued that the LSS has the potential to reshape the health care industry; similarly, it contributed to the automotive industry. The US state hospital of Massachusetts was the first healthcare organization that adopted the Six Sigma mechanism [30]. The Six Sigma black belt certified consultants of GE have facilitated the transformational process and successfully enhanced the radiology department's productivity by 33 percent, followed by the 21.5 percent reduction in the cost. Some other health care centers from Ohio, West Virginia, and Louisiana have also benefited from LSS in the US [31],[32]. The Red Cross hospital of Beverwijk was the first hospital that adopted Six Sigma outside the US, followed by the institute of business and industrial statistics in Amsterdam, Netherlands, which was accountable for 1.2 dollars of saving [33]. The National Health Service (NHS) center of the UK was the first health care organization that adopted both lean and Six Sigma [34]. The successful implementation of LSS will reduce the bottleneck from the process [35], optimizes inventory retention, and reduces the carrying and holding cost of inventory [36]. Nowadays, the LSS project has been implemented in various health care settings such as radiology, inpatient and outpatient diagnostic, surgical procedures, and interventions [37],[38]. Apart from medical procedures, LSS also facilitates administrative management, including medical record-keeping, finance management, patient hospitalization, and discharge forms, and medical equipment coding [39].

## 3. Case study

The XYZ hospital is a tertiary care hospital with a 140-bed facility in Karachi, Sindh. It is specialized in Hematology, Bone Marrow Transplantation, and Allied Surgical/Medical Specialties. In the outbreak period of COVID-19, the passive immunization process came as a savior in the absence of a vaccine. The “loaned” antibodies help prevent certain infectious diseases. The protection offered by passive immunization is short-lived, but it helps protect right away. Vaccines typically necessitate time (weeks or months) to generate protective immunity in an individual to achieve optimum protection. Passive immunization, which is quick-acting, producing an immune response within hours or days, faster than a vaccine.

Moreover, it can reverse a deficient immune system, which is especially helpful in someone who does not respond to immunization. The major hindrance is its cost and harvesting. One of the renowned Hematologists of Pakistan expressed that this technique will be executed via blood plasma from a recovered patient from COVID-19 and introduced into the blood of a patient currently suffering from the coronavirus infection. The injected plasma then produces antibodies and ultimately fights off the virus.

This paper deals with the implementation of DMAIC after one month into the passive immunization process implemented at XYZ hospital. The patients and staff raised many issues that need to be addressed in terms of quality improvement. The following heads cover the DMAIC process to improve the quality of Passive immunization and other departments at this hospital:

### 3.1. Define

The main objective of this phase is to identify the characteristics which are critical to quality (CTQ) to the customer. It is a phase that deals primarily with defining the project teams' role, the project scope and boundary, the customer's voice and expectations, and the high-level goals of the project [40]–[42]. The project revolves around improving the quality of processes after one month into the passive immunization period at XYZ hospital. The primary purpose of this phase is to point out:

1. The reason for low-patient inputs of bone marrow transplant and blood donation for blood disorders.

2. The reason for patient dissatisfaction due to COVID-19.

3. The bottlenecks even after implementing two improvements. (Separate entry and exit points and hiring contractual consultants for passive immunization).

Due to time-constraint to implement the passive immunization project, the hospital merged the OPD, IPD, and PID (Passive immunization department) on a single floor, known as a common facility in the paper, and tried to accommodate all patients through limited resources. The initial improvement steps were not sufficient as the patient input of the core processes was lower than the regular days. Most importantly, word-of-mouth was created that the hospital was treating all kinds of patients under a shared facility that can be dangerous for the hospital brand. So, in this DEFINE stage, the hospital management took the initial improvements of 3-weeks as a starting point. Now, after one-month into the passive immunization, we are applying DMAIC to improve our process. In the defined stage, management uses the following techniques to view the current situation from a bird's eye.

#### 3.1.1. Voice of customer

**Figure 1.**
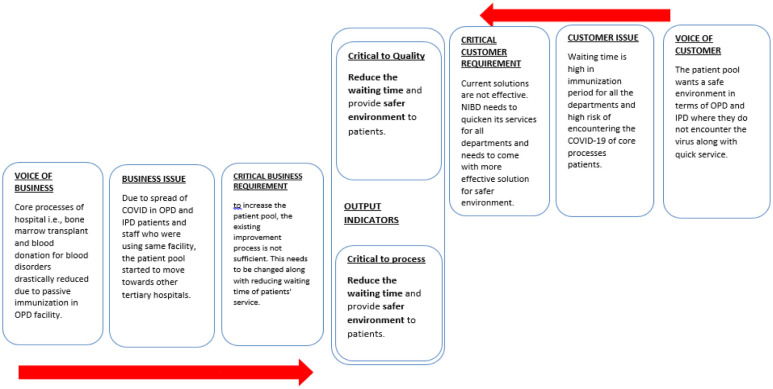
Voice of customer and voice of business.

The first technique employed to capture the customers' voice and perception is the VOC technique. This is a technique that defines what the customer wants from the project and drafts priorities according to their needs, and serves them through the project [43]. Figure 1 depicts how the Voice of Business (VOB) intersects with the Voice of its customers (VOC), which are gathered through informal surveys and observations at the facility. The result shows the two output indicators that are similar for both the business and customer and will serve as the CTQ parameter in the paper. The CTQ are:

1. Reduce the waiting time.

2. Provide a safer environment in the pandemic.

#### 3.1.2. Copis

**Figure 2.**
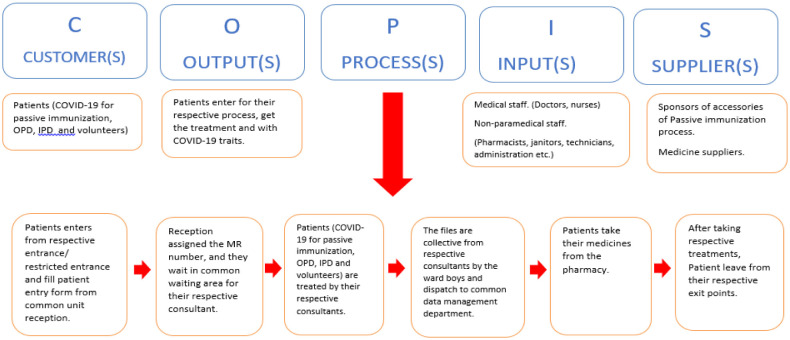
COPIS process diagram of project.

The second technique applied to the CTQ parameters is COPIS. It helps management to map the high-level process which needs to be improved. The COPIS Process map, also known as the SICOP process map, has been used to illustrate the current flow represented in Figure 2. SIPOC is a structured approach used to identify the Process input suppliers, output, and customers [44]. Some pointers were identified through the COPIS, which can serve as a red flag in this process.

- The patients of OPD and IPD have separate entry/exit points as compared to PID patients. However, due to shared facilities, this solution is ineffective in controlling the crowd and providing a safer environment against COVID-19.
- All the departments have a common reception and waiting area.
- The non-paramedical staff is common.
- The data management department is common.

These pointers can serve as a base for brainstorming in later stages. The analysis of both the techniques is combined and moved into the third technique of the Define Phase.

#### 3.1.3. ARMI and communication plan

The ARMI chart defines the roles and responsibilities of all the stakeholders in this process improvement. It is essential to decide who will be responsible for what in which phase and who will ultimately be accountable if the actual work varied from the baseline. It also represents the level of authority and involvement of each stakeholder in each phase of DMAIC. Table 1 represents the key stakeholders responsible for process improvement.

**Table 1.** ARMI chart.

Key stakeholders and team members	Project phases				
	Define	Measure	Analyze	Improve	Control
Medical director	A/I	A/I	A/I	A/I	A/I
Administrative department	I	I	I	I	I
Executives (HODs)	A/I	I	A/I	A	I
Paramedical staff	R/M	R/M	R/M		
Non-paramedical staff	R/M	R/M	R/M		
Research department			R	R	M/R
Regulatory bodies				I	I
Sponsors	I	A/I	I	A/I	I
Project manager	R/M	R/M	R/M	R/M	R/M

After finalizing the roles of each stakeholder in each phase, we defined their level of communication in Table 2. It is essential to have a clear idea of the communication and information flow process in this initiative:

**Table 2.** Project communication plan.

Event	Message	Audience	Frequency	Responsibility	Medium
Project team meeting	Issues/bottlenecks of the process discussed.	HODs, PM, Paramedical and non-paramedical staff	Weekly	PM	Face-to-Face
Status review	Tracking and monitoring the project baselines with the actual schedule and implementation.	PM, HODs, Administrative Department	Bi-monthly	PM	Face-to-Face and email.
Improvement reviews	The process improvements status and new issues counter in its implementation are discussed.	PM, HODs, Administrative Department, Consultants.	Monthly	Administrative and HODs	Face-to-Face
Stakeholder engagement updates	High level view towards the progress of the project improvements.	Medical Director, regulatory bodies, sponsors, HODs, PM and administrative Department.	quarterly/semi-annually	Administrative Department.	Face-to-Face/virtual + Email

Through these techniques, we have identified the areas which needs improvements. But these areas are still at a higher level which needs further brainstorming in the next phase.

### 3.2. Measure

This stage involves picking up the measurement factors which need to be improved [45]. It provides a structure to analyze the current performance of the variables and simultaneously assess, compare, and monitor the improvements along with their capability [46]. The following tools were used to measure the improvement areas mentioned in the defined phase:

#### 3.2.1. Cause and effect diagram

**Figure 3.**
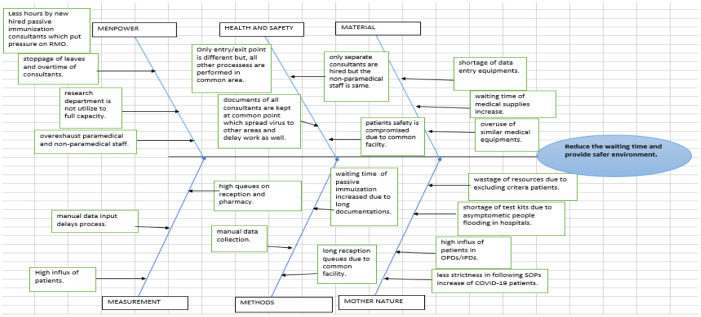
Cause and effect analysis.

The cause-and-effect diagram (popularly known as the Ishikawa diagram) helps the team brainstorm different areas where the problem has occurred. In the case scenario, the whole project management team, administrative department, and the HODs (who could attend) joined the brainstorming session to figure out different areas. The waiting time was increased, and the environment was not safe in a pandemic situation. The Ishikawa diagram is a systematic technique that digs deep to find out the root causes of the problems [47] and provides a relationship between the problem and the identified root causes [45]. There are normally five categories used in the diagram: manpower, machinery, measurement, material, and method. To dig deep into the root cause of the high waiting time and safe environment, we construct a cause-and-effect diagram in Figure 3 of this project:

With the help of this diagram, we find out many causes of the stated problems at XYZ hospital and brainstorm more to pick the important ones.

#### 3.2.2. Data collection plan

The following Table 3 shows the selected X's that we will consider for our further analysis to improve the quality measures at the hospital.

**Table 3.** Data collection plan.

Measure name	Measure type (Y OR X)	Data type (Continuo US/Discrete)
Reduce the waiting time and provide safer environment	Y	
Medical equipment	X	Discrete
Common facility	X	Discrete
Documents	X	Discrete
High influx of patients	X	Discrete
long queues	X	Discrete
Manual data collection	X	Discrete
Data entry equipment	X	Discrete
Common non-paramedical staff	X	Discrete

We move this measured data into the analysis phase where these X's will be evaluated in terms of respondents of the survey.

### 3.3. Analyze

The customer satisfaction-based Kano model was proposed in 1984 by the Japanese professor Noriaki Kano and his team. This model divides product attributes into three categories: threshold or must be, performance, and excitement or delighter. The Kano model is used to determine the customer expectations regarding a service or a product and is used to analyze customer needs and requirements. This model can be used in different ways, depending on the matter in focus. However, it is crucial always to provide the three-category view of the customer regarding the matter in focus. Once it can be used as a model for meeting the features and properties that the service or a product should have, it can be used to define and benchmark the product's basic quality.

The effective approach for business-related decisions can be expected through efficiently gathering data and connecting this data with statistical analyses. Through market research and surveys, important information can be gathered that can be used to identify opportunities, problems, and how to develop effective actions to satisfy customers. There are many different approaches, but one way to get insights from customers is to measure customer satisfaction. Customer opinions are often sought in the form of surveys asking questions about perceptions of quality, experiences, likelihood to come back, and tell friends/relatives about their experience.

We surveyed for process improvement to find out which customers are satisfied or dissatisfied with the hospital services. One of the models to measure customer satisfaction is the Kano model of customer satisfaction and dissatisfaction. We classify the service quality of hospitals during pandemic and passive immunization periods based on how they are perceived by Patients/Attendants/Paramedical staff/Non-Paramedical staff, and their level of satisfaction. The purpose of this survey is to find out how the hospital can bring healthcare improvements by using the Kano model. Total 147 responses were recorded through a survey in which Patients, patient's relatives, healthcare professionals actively participated. This study elaborates on a hands-on way of applying the Kano model based on a view of hospital's services to improve overall healthcare facilities. This research of customer satisfaction influences the improvement of quality management and, in general, the hospital's performance.

#### 3.3.1. Interpretation

According to the Kano model, factors that have a high stated and low derived importance are the least expected factors (must be attributed). Based on the survey Common Facilities, Long queues, and a High influx of patients are highly significant factors for hospital's process Improvement. Hospital Management should focus on these factors and take necessary actions to improve overall healthcare services. Manual Data Collection, Data Entry Equipment, and common non-paramedical staff are moderately significant factors for process improvement. If the hospital management finds them important, they should pay attention and bring necessary changes to these factors. Medical Equipment and documents are the least significant or non-significant factors required for Process Improvement, so hospital management should not consider them or pay much attention when taking necessary actions for process improvement. We not only consider the coefficient values (because their % was too low) but also consider which category got the highest responses.

The below Table includes all the factors for the hospital's process improvement and participants' responses in terms of Kano model attributes.

**Table 4.** Kano evaluation table.

Requirements	A	O	M	I	R	Q
Common facility	1		1	15	128	2
Medical equipment	1	1	1	54	90	
Long queues	1	1	1	6	138	
High influx of patients	1	1		8	137	
Manual data collection	1		1	30	114	1
Data entry equipment		1	1	26	119	
Common non-paramedical staff	2		1	25	118	1
Documents	1			50	96	

The reverse factor is important here as all these variables who have high reverse value are perceived negatively by the customers. The ranges of importance are:

1. 1–65% = non-significant.

2. 66–85% = moderately significant

3. 85 above = highly significant.

**Table 5.** The classification of attributes according to Kano model.

									Customer satisfaction coefficient	
Product feature	A	O	M	I	R	Q	Total	Category	Extent of satisfaction (A+O)/(A+O+M+I)	Extent of dissatisfaction −(O+M)/(A+O+M+I)
Common facility	1/0.68%		1/0.68%	15/10.20%	128/87.07%	2/1.36%	147	R	0.058	−0.058
Medical equipment	1/0.68%	1/0.68%	1/0.68%	54/36.73%	90/61.22%		147	R	0.035	−0.035
long queues	1/0.68%	1/0.68%	1/0.68%	6/4.08%	138/93.8%		147	R	0.22	−0.22
High influx of patients	1/0.68%	1/0.68%		8/5.44%	137/93.19%		147	R	0.2	−0.1
Manual Data collection	1/0.68%		1/0.68%	30/20.40%	114/77.55%	1/0.68%	147	R	0.03	−0.03
Data entry equipment		1/0.68%	1/0.68%	26/17.6%	119/80.95%		147	R	0.035	−0.07
Common non-paramedical staff	2/1.36%		1/0.68%	25/17.00%	118/80.27%	1/0.68%	147	R	0.07	−0.035
Documents	1/0.68%			50/34%	96/65.30%		147	R	0.019	0

**Table 6.** The satisfaction index by the presence of an attribute (in order of strength of satisfaction).

Y	X's	Kano (%)		Category (%)	Results
		Extent of satisfaction	Extent of dissatisfaction		
Increase the Patient Pool of Core Processes	Common Facility	0.08	−0.07	R (87.07%)	Highly significant
	Medical Equipment	0.035	−0.035	R (61.22%)	Non-significant
	Long Queues	0.22	−0.22	R (93.87%)	Highly significant
	High influx of patients	0.2	−0.1	R (93.19%)	Highly Significant
	Manual Data collection	0.03	−0.03	R (77.55%)	Moderately- Significant
	Data entry equipment	0.035	−0.07	R (80.95%)	Moderately significant
	Common non-paramedical staff	0.07	−0.035	R (80.27%)	Moderately significant
	Documents	0.019	0	R (65.03%)	Non-significant

### 3.4. Improve

This is the phase where all the team gathers to their work into action. The solution has been identified in the previous phases, action plan is devised, and implementation is now having to be carried out to reduce the variation and improve the target performance [48]. This is the phase where the root cause of the problem is visible that helps in coming up with range of solutions or countermeasures, commonly through documentation review and expert opinions.

Addressing the root cause is the right start to finding out possible range of solutions to eliminate the root cause. The issue in discussion of this paper was thoroughly analyzed and defined in the above phases and through brainstorming and the method that was used was Kaizen Events integrated with the DMAIC improve phase. This method is very efficient when working out and implementing solutions on narrow projects by a combined effort, experience, and brainstorming of the team [48].

#### 3.4.1. Kaizen approach

In this paper, Kaizen method was used to find the root cause and gather range of solutions by using the three steps of implementation approach namely, 1. Open Meeting, 2. Kaizen Activities, 3. Close Meeting. The management, doctors, and other team members work together through the process and figure out which kaizen activities can be improved. Communication is the key here.

It must be mentioned that the most important aspect of the kaizen even approach is the focus on the customer. This can be done by seeking and working out the Critical to Quality (CTQ) requirements and the core business processes that will help with the implementation of the CTQs [49]. The three-step approach was integrated in this paper to carry out the kaizen approach. Below is the table that briefly shows the process of three step of implementation approach.

**Table 7.** Three steps of implementation approach.

Step	1	2	3
Topic	Open Meeting	Kaizen events	Close Meeting
Event	Meeting is called to order to discuss the process Existing status of the survey for customer satisfaction	To find problem roots. 5 Why Technique. Implementing the Kaizen project	Standardization maintaining Kaizen report ppt data

- **Step 1-Open Meeting:** A meeting is called in order by the management and basic intro is given on using the tools and the skills required.
- **Step 2-Kaizen Activities:** The team workout the problem and find range of solutions. This paper discussed the use of 5 Why to brainstorm the root causes and find possible solution. This will be discussed later in this paper. Possible solutions were discussed to reduce the waiting time and provide safer environment.
- **Step 3-Close Meeting:** At the end, a close meeting is scheduled. The team present the solution implementation, the process, and achievements. The results are analyzed, and final decision is made by the management/project manager whether the solution was satisfactory or not.

#### 3.4.2. Kaizen activity: root findings—5 why approach

In this project, the 5 Why Approach was used to find the root causes and analyze them to find possible range of solutions. This was carried out by the Cross Functional Teams (CFT). This is a comprehensive and strategic process where the members brainstorm the root cause of the problems and provide solutions both backed up by data and facts [50].

The CFT team carried out this activity to address the problem to reduce the waiting time and provide safer environment. Brief of the approach discussed above is summarized below:

**Table 8.** Summary of 5 why approach.

X	Why 1	Why 2	Why 3	Why 4	Solution
Common Facility	limited time available for implementation	COVID-19 cases were increasing rapidly.	Quick solution to the virus.	No vaccine- increasing risk overall patient pool	Shift OPD and IPD on first floor and keep ground floor specific for passive immunization.
Data entry equipment's	overload from multiple departments	High number of patients	Dedicated single facility for multiple processes.	Time constrained	Involve research department in it which will increase equipment's.
Common non-paramedical staff	shortage of staff	Overload of work	High number of patients		Hire contractual non-paramedical staff for passive immunization.
High influx of patients	Core processes patients along with COVID-19.	Time constrained	React quickly to increasing number of patients.		Change department's locations and all its related processes which will divide the influx.
Long queues	Higher number of patients	Common facility	Time-constraint		Adopt Teleclinic system through which consultations of OPD can be on call which can reduce queues.
Manual Data collection	Less number of computers	It was only for OPD before this project			Incorporate research department in data collection process

The paper discussed six common factors that can possibly affect the increase of patient pool in core processes leading to the dissatisfaction of the customers and creating an unsafe environment. The factors are as below:

1. Common Facility—Because of the pandemic in full swing, the implementation of the project was a challenge, especially with the rise in cases and deaths day by day. Because of this, there was limited time, and a quick solution to eradicate the virus was everyone's concern.

2. Data entry equipment's—Because of the huge crowd coming in due to pandemics and facilities and staff being in the same facility created a considerable delay in processing the requests of the customers/patients. Manual data entry also resulted in delays.

3. Common non-paramedical staff—Same facility, same staff, and the huge influx of patients were challenging for the staff.

4. The high influx of patients—The hospital initially decided to use the same facility for OPD, IPD, and PID. Because the hospital core processes were being greatly affected by using the same vicinity for COVID-19 patients.

5. Long queues—Because of the above factor, time management and response time were challenging and affected customer satisfaction.

6. Manual Data collection—The facility was non-existent for the PID and IPD before this project. This was a challenge, especially during the influx of patients during the pandemic.

#### 3.4.3. Prioritizing the solutions

After the 5 Why approach, the team decided to prioritize the range of solutions by analyzing them their level of ease, cost and impact on the factors identified. Findings from this activity is given below while highlighting their importance and priority.

**Table 9.** Prioritizing solutions.

Action	Ease (8)	Cost (10)	Impact (9)	Total	Importance
Shift OPD facility to first floor	4	5	5	127	P1
involve research department	4	3	3	89	P2
hire contractual non-paramedical staff	1	2	3	55	P4
adopt Teleclinic system for OPD	2	3	4	82	P3

Note: ease: 1–5, 1 is easiest; cost: 1–5, 1 is lowest; impact: 1–5, 1 is lowest. Scoring—(Action 1: will have a huge impact, with moderate level of effort and scored moderate on ease which may drag the cost high. Action 2: will have moderate impact and scored moderate on ease with equal effect on the cost. Action 3: will similarly have moderate impact with low impact on the ease and cost. Action 4: will have moderate impact with low level of ease and cost.)

### 3.5. Control

This is the last phase in the DMAIC phase. This phase ensures that the action plan created in the improve phase is well implemented and followed through while maintaining close eye on the development [51]. The management ensures that proper execution of the initiatives is being carried out and the development and effectiveness are also evaluated.

In this paper, following controlling plan was developed for identifying the major areas and players in carrying out this phase successfully. Summary is shown below:

**Table 10.** Implementing solution and controlling.

What is implemented	Where it is to be implemented	WHO will implement	By when	How it is to be implemented	Frequency of check	Checked by
Shift OPD facility to first floor	Hospital main branch	PM	1^st^ May–15^th^ May	slowly moving consultants and OPD/IPD equipment's to first floor	weekly	admin. department
Involve research department	Hospital main branch	PM	1^st^ May–10^th^ May	Get aware with the current work process of the Research department and draft a quick plan to incorporate them for data collection and equipment's.	weekly	admin. department
Adopt Tele-clinic system for OPD	Hospital main branch	IT department	5^th^ May–10^th^ May	Develop an app and share on website of xyz hospital.	daily	PM
Hire contractual non-paramedical staff	Hospital main branch	Administration Department	3^rd^ May–10^th^ May	Publish in newspaper and on websites.	daily	PM

This is how we can improve the quality of the process through DMAIC at hospital. We can even use poke-yoke as a controlling measure where we assign color-oriented tags to each department patients/staff and track it through the system if anyone enters the other or unauthorized department.

## 4. Conclusions and future recommendation

DMAIC methodology is a strategic technique to improve the project performance by implementing lean Six Sigma process improvement through effective implementation of both statistical and mathematical tools. The DMAIC model presented in this case study provides an organized, recurrence, and systematic approach for identifying, analyzing, improving, and controlling the hospital management process. It allows healthcare managers to tailor the awareness-raising efforts to specific working conditions that directly serve urgent needs. The study provides the integration of different LSS tools aligned with the process of DMAIC to solve the operational complications of the hospital. The structured approach will help in achieving short-term objectives, but it also provides direction for the formulation of long-term policies. Initially, the problem was defined by interpreting the Voice of the Customer and voice of the business to identify the CTQ's of the project. Once the problem was defined, the role and responsibilities were assigned to the project team by using ARMI chart. The measuring process was carried out by implementing fishbone diagram and filtered using a data collection plan. The critically identified variables were analyzed using the Kano model. The 4w1h techniques followed by the 5why root cause analysis were performed to improve and control the project objects. Other industries could use this set of tools to evaluate and optimize routine problems, ultimately enhancing the quality and reducing cost. The Healthcare industry is one of the most critical sectors of our society. In the future, the researcher can conduct a more in-depth analysis for process and quality improvement. PDCA (Plan, do, check and act), DMADV (design, measure, analyze and improve), JIT (just in time), QFD (quality Function Development), FMEA (Failure mode and effect analysis) and many other quality improvement methodology can be adopted for enhancing the hospital quality management system.
